# Clinical Patterns of Traditional Chinese Medicine for Ischemic Heart Disease Treatment: A Population-Based Cohort Study

**DOI:** 10.3390/medicina58070879

**Published:** 2022-06-30

**Authors:** Lung-Shuo Wang, Pei-Tzu Yen, Shih-Feng Weng, Jong-Hau Hsu, Jwu-Lai Yeh

**Affiliations:** 1 Department of Chinese Medicine, Sin-Lau Hospital, Tainan 70142, Taiwan; lonsuo@gmail.com (L.-S.W.); agneskyetabo@gmail.com (P.-T.Y.); 2 The School of Chinese Medicine for Post Baccalaureate, I-Shou University, Kaohsiung 82445, Taiwan; 3 Department of Healthcare Administration and Medical Informatics, Kaohsiung Medical University, Kaohsiung 80708, Taiwan; sfweng@kmu.edu.tw; 4 Graduate Institute of Medicine, College of Medicine, Kaohsiung Medical University, Kaohsiung 80708, Taiwan; 5 Department of Pediatrics, College of Medicine, Kaohsiung Medical University, Kaohsiung 80708, Taiwan; 6 Department of Pediatrics, Kaohsiung Medical University Hospital, Kaohsiung 80708, Taiwan; 7 Department of Medical Research, Kaohsiung Medical University Hospital, Kaohsiung 80708, Taiwan; 8 Department of Pharmacology, College of Medicine, Kaohsiung Medical University, Kaohsiung 80708, Taiwan

**Keywords:** traditional Chinese medicine, ischemic heart disease, prescribing patterns, national health insurance database, Taiwan

## Abstract

*Background and objectives:* Traditional Chinese medicines (TCMs) are widely prescribed to relieve ischemic heart disease (IHD); however, no cohort studies have been conducted on the use of TCMs for patients with IHD. The aim of the study was to analyze TCM prescription patterns for patients with IHD. *Materials and Methods:* The retrospective population-based study employed a randomly sampled cohort of 4317 subjects who visited TCM clinics. Data were obtained from the National Health Insurance Research Database (NHIRD) of Taiwan for the period covering 2000 to 2017. Data analysis focused on the top ten most commonly prescribed formulae and single TCMs. We also examined the most common two- and three-drug combinations of TCM in single prescriptions. Demographic characteristics included age and sex distributions. Analysis was performed on 22,441 prescriptions. *Results:* The majority of TCM patients were male (53.6%) and over 50 years of age (65.1%). Zhi-Gan-Cao-Tang (24.76%) was the most frequently prescribed formulae, and Danshen (28.89%) was the most frequently prescribed single TCM for the treatment of IHD. The most common two- and three-drug TCM combinations were Xue-Fu-Zhu-Yu-Tang and Danshen” (7.51%) and “Zhi-Gan-Cao-Tang, Yang-Xin-Tang, and Gua-Lou-Xie-Bai-Ban-Xia-Tang” (2.79%). *Conclusions:* Our results suggest that most of the frequently prescribed TCMs for IHD were Qi toning agents that deal with cardiovascular disease through the promotion of blood circulation. The widespread use of these drugs warrants large-scale, randomized clinical trials to investigate their effectiveness and safety.

## 1. Introduction

Ischemic heart disease (IHD), also known as coronary heart disease, is a leading cause of death and disability worldwide. According to global statistics, the total number of IHD cases reached 182 million in 2019, resulting in roughly 9 million deaths [1]. The World Health Organization listed IHD as number one in the global burden of disease in 2020. Statistics published by the Ministry of Health and Welfare, Taiwan, in 2020 listed chronic diseases such as cancer, heart disease, and pneumonia as the top three causes of death. Heart disease was a major cause of death among individuals over 45 years of age, and the number of deaths due to heart disease is increasing. Classical symptoms of IHD include chest tightness and chest pain, the onset of which can be sudden (i.e., without previous symptoms). Treatment for IHD focuses on the selection of appropriate medications and mediating risk factors, including high cholesterol, high blood pressure, diabetes, poor kidney function, metabolic syndrome, obesity, imbalances in diet, a lack of exercise, smoking, and stress [2]. In severe cases, cardiac catheterization and stent placement are necessary. In cases where the vascular obstruction is too severe, coronary artery bypass surgery may be performed [3].

IHD has been known since ancient times. Practitioners of traditional Chinese medicine (TCM) have thousands of years of experience in the treatment of IHD, and TCM has remarkable clinical efficacy. Evidence-based research into the effects of traditional herbs and prescriptions begins with an analysis of prescription patterns using advanced statistical methods. Many TCMs are aimed at promoting blood circulation and minimizing blood stasis in order to reduce the incidence of angina pectoris and protect the heart. Pharmacological studies have confirmed that many active ingredients in TCMs can indeed stabilize heart rhythm, increase coronary blood flow, and reduce heart palpitations. Some recent findings point to the potential application of TCM in the treatment of IHD, such as the myocardial therapeutic effect of the “Danggui–kushen herb pair” achieved by inhibiting NF-*κ*B and HIF-1*α* signaling [4] as well as the post ischemic myocardial inflammation amelioration of “Compound Danshen Dripping Pills” accomplished through synergistically regulating MAPK, PI3K/AKT and PPAR signaling pathways [5].

Previous cohort studies have examined the use of TCM to treat coronary diseases between 2000 and 2011 [6,7,8]. In the current study, we explored TCM prescription patterns from 2000 to 2017, using the population-based NHI database in Taiwan. Searches were conducted to determine the number and frequency of visits to TCM clinics under diagnostic codes specific to IHD: ICD9 410–414 (ICD10: I20–I25). We then ranked frequently prescribed TCM formulas and single TCMs for IHD. These results provide a valuable reference for clinical treatment of patients with IHD.

## 2. Materials and Methods

### 2.1. Data Sources

The National Health Insurance Administration (NHIA) provides researchers with encrypted data pertaining to prescriptions and treatment details, medical orders, hospitalization expenses, and other data files. In Taiwan, TCM medical activities are restricted to ambulatory clinics, and only licensed TCM doctors are reimbursed by the NHI. The current statistical research was based on complete TCM ambulatory care claims data from the NHI Research Database for the period 2000 to 2017. The TCM ambulatory care claim data were then linked with files including the details of ambulatory prescriptions excluding inpatient care claims data. All data collection was performed under the approval and supervision of the Institutional Review Board (IRB) at Kaohsiung Medical University Hospital [IRB number: KMUHIRB-E(II)-20200027; approval dated: 30 March 2020].

### 2.2. Study Design

In Taiwan, TCM doctors make diagnoses in accordance with ICD-9-CM coding. In the current study, we focused exclusively on patient data with the diagnosis code for IHD. We then sorted the 4317 subjects who visited TCM clinics for IHD. This led to the retrieval of 22,441 prescriptions for TCM. Our database included six interlinked variables: month and year of the fee, application type, hospital code for hospital identification, application date, case type, and serial number. Based on the theory of Chinese medicine, there are four types of TCM prescriptions: (1) a single herb; (2) a composition of multiple herbs with various dosages (Fu-Fang); (3) a regimen of herbs with fixed dosages in line with classical or well-known TCM textbooks (Fang-Ji); and (4) a classical formula in which a drug compound is combined with herbs (Chia-Chien-Fang). Note that combinations of ingredients are in powder form, which facilitates mixing within a single prescription.

### 2.3. Statistical Analysis

All IHD subjects were defined by the diagnosis code ICD9 410–414 (ICD10: I20–I25). Age and gender-specific frequencies for the use of TCMs among patients with IHD from 2000–2017 were examined. We used SAS 9.4 (SAS Institute, Inc., Cary, NC, USA) to link ambulatory care claim data and data with the details of ambulatory prescriptions. Frequency and patterns of TCM formula or single herbs were performed by statistical software R, which is a publicly available software in our institution.

Association rules and frequent itemset mining are well-established approaches to discovering specific relationships among variables in large databases. Data mining was first developed in the 19th century [9] and has been widely applied in medical research over the last few decades [7,8]. In the current study, data mining was used to characterize TCM prescription patterns for IHD. Parameter X, the support (%) of a prescribed drug set, was defined as the group of the data set comprising drug set X. Here, support is defined as the frequency with which a rule appears in the database. Using statistical software R (version 2.13.2, The R Foundation, Vienna, Austria) package ‘arules’, we used the function ‘apriori’ with minimum support set at 1%.

## 3. Results

### 3.1. Patient Features

**Figure 1:**
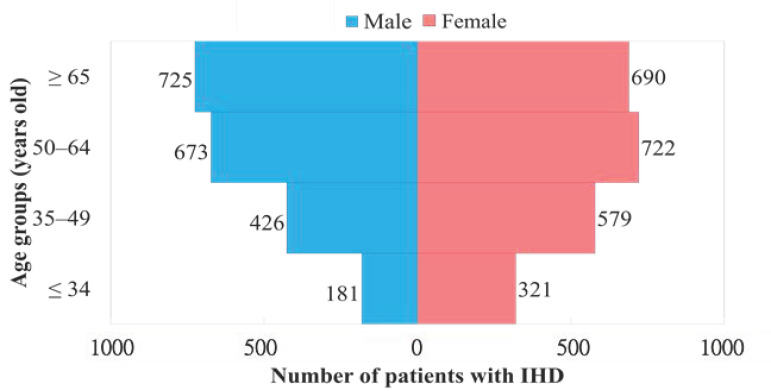
Relationship between the number of patients with ischemic heart disease (IHD) and age groups (total patient number, *n* = 4317).

Using the screening diagnosis code ICD9 410–414 (ICD10: I20–I25), we identified 4317 patients who sought TCM treatment for IHD. The patients were distributed according to age into four categories: ≤34, 35–49, 50–64, and ≥65 years. Gender classification was based exclusively on physical gender (regardless of psychological gender). As shown in Table 1, the number of consultations for IHD increased with age, likely due to a gradual deterioration of heart function with age. Overall, consultations for IHD increased from the age of 35, and individuals ≥ 50 years accounted for 65.1% of the cases of morbidity. In terms of gender differences (Figure 1), the incidence of morbidity among females exceeded that of men prior to the age of 49 (earlier onset), whereas the incidence of morbidity among males was 1.14 times that of women after 50 years of age (higher overall incidence).

### 3.2. Single TCMs

As shown in Table 2, Danshen (28.89%) was the single TCM most commonly prescribed for IHD, followed by Xiebai (24.76%), Yujin (21.30%), Honghua (17.25%), Huangqi (9.98%), Gualouren (9.54%), Yanhusuo (7.99%), Chuanxiong (7.09%), Gegen (6.66%), and Danzhuye (6.18%). Table 3 lists the traditional use and evidence-supported single TCMs for IHD.

### 3.3. TCM Formulas

As shown in Table 4, Zhi-Gan-Cao-Tang (24.76%) was the TCM formula most commonly prescribed for IHD, followed by Xue-Fu-Zhu-Yu-Tang (roughly 20%) and Sheng-Mai-Yin (roughly 20%), Gua-Lou-Xie-Bai-Ban-Xia-Tang (9.98%), Tian-Wang-Bu-Xin-Dan (9.54%), Yang-Xin-Tang (6.66%), Zhen-Wu-Tang (5.78%), Jia-Wei-Xiao-Yao-San (5.34%), Fufang-Danshen-Pian (4.46%), and Ji-Sheng-Shen-Qi-Wan (4.03%). Table 5 lists the traditional use and evidence-supported TCM formulas for IHD.

### 3.4. TCM Combinations

**Figure 2:**
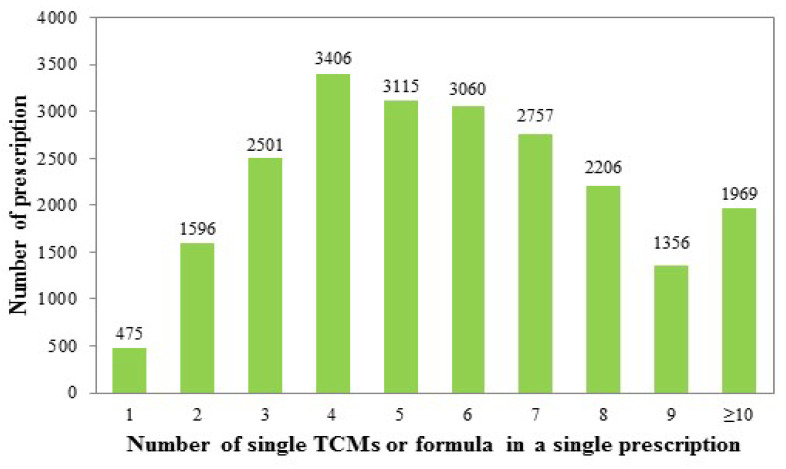
Relationship between the number of prescriptions and the number of TCMs. The average is 5.82 ± 2.77 TCM items in a single prescription for treatment of subjects with IHD (total prescription number, *n* = 22,441).

Ten TCMs (singly or in formulas) accounted for roughly 15% of the prescriptions. Most single prescriptions contained 5 to 7 items (average of 6.15) (Figure 2). The most common two-drug combination was Danshen plus Bu-Yang-Huan-Wu-Tang (Table 6). The most common three-drug combination was Bu-Yang-Huan-Wu-Tang plus Shichangpu plus Yuanzhi (Table 7).

## 4. Discussion

In ancient TCM texts, the terms used to describe diseases related to IHD included “Xin Tong” (heartache), “Xin Jue” (heart syncope), “Zhu Xin Tong” (acute heartache), and “Zhen Xin Tong” (true heartache). In the oldest ancient pharmacopeia “Huangdi Neijing” (The Yellow Emperor’s Inner Canon, published in China, 99-26 B.C.), there are detailed descriptions of IHD: “For the true heartache, if your hands and feet become blue and reach your elbows and knees, the pain is severe. It usually occurs in the morning and fades in the evening, or it occurs in the evening and fades in the morning”. These descriptions of pain in the precordial area were attributed to poor qi and blood flow. In some cases, they mentioned pain in the shoulders and back, shortness of breath and wheezing, and an inability to lie down, all of which fell within the designation of “heartache”. These descriptions are similar to the clinical manifestations of coronary heart disease and angina pectoris.

In Western medicine, most diagnoses of coronary heart disease are based on the results of angiography or computer tomography. Due to the high risk of mortality, treatment often involves highly invasive emergency procedures. TCM is meant to prevent coronary artery obstruction and disease using non-invasive methods [41]. This may also explain why we recorded 22,441 prescriptions for only 4317 subjects during the 18-year study period.

The most frequently prescribed single TCM was Danshen (dried root of *Salvia miltiorrhiza*), which possesses a bitter taste and cold nature associated with the heart and liver meridians. It was classified as a top-grade medication in “Shennong Bencao Jing” (Shennong’s Classic of Materia Medica, published in China, 96-31 B.C.) for the treatment of heart and spleen disease, to clear the blood circulation system, relieve irritation, and replenish the Qi. Danshen has also been recommended for the treatment of angina pectoris in “Yi Xue Xiao Jin Zhen” (Medical Golden Needle, published in China, 1916). A number of recent scientific studies have examined the cardioprotective effects of Danshen (in crude form, in specific preparations for injection, or in pill form) [42]. The constituents from Danshen include lipophilic components (tanshinone IIa and cryptotanshinone) as well as hydrophilic compounds (Sal-A and Sal-B), which have exhibited beneficial effects for the heart due to their anti-inflammatory, anti-oxidant, and anti-apoptotic properties [43,44,45,46]. Danshen is widely prescribed for the treatment of cardiovascular diseases, such as coronary heart disease, myocardial infarction, atherosclerosis, and angina pectoris [47,48,49,50]. 

Another frequently prescribed single TCM was Yujin (*Curcumae Longae* Radix), which is the dry root of *Zingiberaceae cultivars*, such as *Curcuma wenyujin*, *Curcuma kwangsiensis*, *Curcuma longa*, and *Curcuma phaeocaulis*. Yujin also possesses a bitter taste and cold nature associated with the heart, gut, and liver meridians. According to the “Bencao Beiyao” (Essentials of Materia Medica, published in China, 1694), Yujin can cool the heart and blood, disperse stagnation, and normalize the gallbladder to cure jaundice. Clinically, it is often used to treat chest, flank, and abdominal pain induced by Qi stagnation and blood stasis. A number of recent scientific studies have reported that Yujin activates blood flow and mediates blood stasis by inhibiting the aggregation of platelets, slowing the over-activation of the coagulation, and promoting blood circulation [14]. Curcumin and curcumin-containing extracts of Yujin have also been shown to promote blood circulation, relieve pain, and protect against myocardial ischemia-reperfusion injury by inhibiting lipid peroxidation, augmenting endogenous antioxidants, and improving myocardial metabolism [15,51,52].

Yanhusuo is the dry tuber of *Corydalis yanhusuo*, which possesses an acrid-bitter taste and a warm nature associated with the heart, liver, and spleen. Traditionally, Yanhusuo has been used to promote blood circulation, invigorate the qi, and relieve pain. The ancient pharmacopeia “Leigong Paozhi Lun” (Master Lei’s Discourse on Drug Processing, published in China, 5th century) recommended the use of Yanhusuo “to treat dying heartache”. The “Bencao Gangmu” (Compendium of Materia Medica, 1593 A.D.) also reported on the therapeutic effects of Yanhusuo in treating blood and qi stagnation as well as pains throughout the body. Recent scientific studies have extracted alkaloids, such as dextrocorydine, tetrahydropalmatine, and protopine, which have been shown to have potent analgesic and antispasmodic effects on the central nervous system. Another alakaloid, l-Tetrahydropalmatine, exhibited cardioprotective effects against acute global cerebral ischemia-reperfusion injury by activating the PI3K/Akt/eNOS/NO pathway and decreasing the accumulation of inflammatory factors, such as TNF-α and MPO [23]. Dehydrocorydaline alkaloid has been shown to ameliorate atherosclerosis in ApoE-/-mice via inflammatory inhibition targeting through macrophage p65- and ERK1/2-mediated pathways [24].

Gegen possesses a flat, sweet taste and has been associated with the Yangming meridian and spleen meridian. Traditionally, it was used to uplift the qi in the stomach, enhance fluid production, and quench the thirst as well as promote sweating and relieve muscles tightness and fever. It has traditionally been used to treat spleen and stomach diseases as well as diarrhea. Recent scientific studies have demonstrated that Gegen can dilate coronary and cerebral blood vessels and increase blood flow. Gegen contains bioactive ingredients (e.g., puerarin) and other flavones and isoflavones, which have been shown to inhibit platelet aggregation and improve the metabolism of the ischemic myocardium [53,54]. A number of studies have reported on the cardioprotective potential of puerarin by reducing myocardial oxygen consumption and increasing coronary oxygen supply [55]. Researchers have also reported that Gegen can reduce the area of myocardial infarction, reduce serum CK-MB activity, and reduce apoptotic cell death [56]. Isoflavones from Gegen have also been shown to have anti-atherosclerosis effects. For example, daidzin and formononetin can lower serum cholesterol while genistein can lower triglyceride levels [57]. A number of studies have confirmed that Gegen possesses cardioprotective, neuroprotective, vasodilatation, anti-oxidation, and anti-inflammatory effects. Other studies have demonstrated the effects of Gegen in preventing coagulation, protecting vascular endothelial cells, and preventing damage due to myocardial ischemia. It has even been shown to enhance insulin resistance and lower blood sugar levels. It is widely used in the treatment of cardiovascular disease and type 2 diabetes [58].

The most popular TCM formula in this study was Zhi-Gan-Cao-Tang, which can be traced back to the ancient pharmacopeia “Shanghan Lun” (Treatise on Cold Pathogenic Diseases, published in China, c.a. 200–210). It has been recommended for the treatment of typhoid fever, pulse congestion, and heart palpitations, owing to the effects on replenishing qi, nourishing yin, and tonifying yang as well as rejuvenating the pulse. The main constituents are from Radix *Glycyrrhizae* and Radix *Ginseng*, glycyrrhizin and ginsenosides, all of which have been shown to improve the myocardial contractility and cardiac function by inhibiting of Na^+^/K^+^−ATPase [29]. They have also been linked to the alleviation of oxidative stress in cardiomyocytes due to toxins or inflammation-associated vascular diseases, such as atherosclerosis [30,31].

The second most popular TCM formula in this study was Xue-Fu-Zhu-Yu-Tang, which can be traced back to the pharmacopeia “Yilin Gaicuo” (Correction of Errors in Medical Classics, published in China, 1830). This combination of 11 herbs is used mainly to promote blood circulation and remove blood stasis or as a supplement to soothe the liver or regulate the qi. Clinical applications include the treatment of blood stasis diseases, such as rheumatic heart disease, coronary heart disease, and angina pectoris, as well as chest contusion, intercostal neuralgia, chest pain of costochondritis, and sequelae of concussion. Pharmacological analysis has revealed that Xue-Fu-Zhu-Yu-Tang has anticoagulant and thrombolytic effects, including the dilation of peripheral blood vessels and coronary arteries to promote blood circulation [59]. In blood stasis and chest pain, Chaihu (*Bupleurum chinense* DC./*Bupleurum scorzonerifolium* Willd.), Chuanxiong (*Ligusticum chuanxiong* Hort.), and Chishao (*Paeonia lactiflora* Pall./*Paeonia veitchii* Lynch) have been linked to analgesic effects, while Chuanxiong, Chishao, Zhishi (*Citrus aurantium* L.), and Gancao (*Glycyrrhiza uralensis* Fisch./*Glycyrrhiza inflata* Batalin/*Glycyrrhiza glabra* L.) have been linked to the alleviation of smooth muscle spasms. Xue-Fu-Zhu-Yu-Tang has been shown to reduce the concentration of triglycerides in serum, decrease the ratio of TXA2/PGI2, and decrease the production of proinflammatory cytokines in rats fed a high cholesterol diet [32]. Xue-Fu-Zhu-Yu-Tang has also been shown to reverse myocardial fibrosis in hypertensive rats by increasing myocardial cell protection and decreasing TGF-β1 mRNA and protein expression [60]. In a swine phlegm and blood stasis type coronary heart disease model, Xue-Fu-Zhu-Yu-Tang combined with Gua-Lou-Xie-Bai-Ban-Xia-Tang was shown to have significant protective effects against myocardial apoptosis, upregulate Bcl-2 protein expression, and downregulate Bax, Caspase-3, and Caspase-9 protein expression [34].

Another famous TCM formula, Sheng-Mai-Yin, can be traced back to the ancient pharmacopeia “Lantai Gueifan” (Lantai Standard, published in China, 1764). This formula, containing Panax ginseng, Ophiopogon japonicus, and Schisandra chinensis, is commonly used to deal with coronary heart disease, angina pectoris, viral myocarditis, heart failure, and other heart diseases. Recent scientific studies have reported that it can also have protective effects on the heart, such as reducing early ventricular contraction and paroxysmal tachycardia, by suppressing the sodium and potassium ATPase pumps of the myocardial cell membrane [29]. Injections based on Sheng-Mai-Yin have been shown to promote the recovery of cardiac performance in patients who underwent bypass grafting of the coronary artery [35].

The TCM formula Gua-Lou-Xie-Bai-Ban-Xia-Tang can be traced back to the pharmacopeia “Jingui Yaolue” (Synopsis of Golden Chamber, published in China, c.a. 205). This modification of Gua-Lou-Xie-Bai-Bai-Jiou-Tang through the addition of Banxia (*Pinellia ternata* (Thunb.) Makino) is frequently prescribed for IHD. This formula contains the cold bitter Gualouren (*Trichosanthes kirilowii* Maxim./*Trichosanthes rosthornii* Harms), which is used to reduce phlegm and relieve chest tightness. It also contains the warm smooth Xiebai (*Allium macrostemon* Bunge), which is used to promote yang qi and activate the blood. Another constituent, Banxia, is used to reduce dampness, limit phlegm production, and halt vomiting. Gua-Lou-Xie-Bai-Ban-Xia-Tang is meant for coronary heart disease involving qi deficiency, phlegm, and blood stasis and is clinically used for chest obstructive syndrome, such as coronary heart disease angina, intercostal neuralgia, arrhythmia, chest soft tissue injury, intercostal chondritis, and emphysema. Recent scientific evidence suggests that Gua-Lou-Xie-Bai-Ban-Xia-Tang could have cardioprotective effects in ischemia-reperfusion models, including the inhibition of NF-κB and inflammatory cytokines [36]. It has also been shown to have protective effects against type II diabetes and acute myocardial ischemia by ameliorating oxidative stress and apoptosis via PI3K/Akt/eNOS suppression [38].

The results of this study could be used as a reference for further in-depth research. Note however that the results of this study in no way attest to the efficacy of these TCMs. Due to the risk of potentially dangerous side effects, these herbs should not be taken without professional consultation or thorough diagnostic procedures. For instance, a few scientific studies have indicated safety concerns for Danshen, such as decreased appetite, abdominal discomfort, convulsions, allergy, and dystonia [61].

This study was prone to inevitable errors, including inaccurate diagnoses. In many cases, patients with chest or heart discomfort receive a diagnosis of IHD (ICD9 diagnosis code: 410–414 and ICD10 diagnosis code: I20–I25) before undergoing a thorough examination. Or, for patients with IHD, only the other forms of heart disease (ICD9-CM diagnosis code 420–429 and ICD10 diagnosis code: I30–I52), chest pain (ICD9-CM diagnosis code 786.5 and ICD10 diagnosis code: R079), and other chest pain (ICD9-CM diagnosis code 786.59 and ICD10 diagnosis code: R0789) were declared in the NHI database, which also caused the error of case number reducing. Another source of error was the stacking of formulas with single TCMs, which increased the proportion of specific herbs. These errors are difficult to eliminate; however, they do not reduce the credibility of the research, due to the fact that these prescriptions were deemed effective by doctors in terms of medical theory and empirical use. In addition, this study was subject to a number of limitations. Identification of the best TCM formula for a specific patient should be based on careful analysis of all clinical information obtained through the four diagnostic methods (inspection, auscultation/olfaction, inquiry, and palpation) and focusing on the eight principal syndromes (Yin, Yang, outer, inner, cold, heat, deficiency, and actuality). We were unable to collect detailed medical records for each patient, and many IHD patients also underwent modern medical treatments, such that the efficacy of the TCMs could not be assessed objectively. Note also that in Taiwan, many Chinese herbal medicines or health foods containing Chinese herbal ingredients can be purchased directly from TCM pharmacies or drugstores, which makes it impossible to assess the effects of inadvertent drug interactions. For example, the combined use of Warfarin and herbal medicines, such as Danshen and Licorice (the major herb in Zhi-Gan-Cao-Tang), can over-activate the anticoagulant effect and lead to bleeding complications [62]. Danshen has also been discovered to synergistically cooperate with other herbal medicines (such as Radix *Puerariae lobatae*, and Danhong injection), or Western drugs (such as Atorvastatin) in the treatment of cardiovascular diseases [61].

## 5. Conclusions

This paper reports on TCM prescription patterns for IHD from the NHI Research Database. The single herb most frequently prescribed for IHD was Danshen (*Salvia miltiorrhiza*), and the most popular formula was Zhi-Gan-Cao-Tang. The safety and therapeutic effects of these TCMs will require further exploration in future studies.

## Figures and Tables

**Table 1:** Age-specific frequencies for the use of traditional Chinese medicines (TCMs) among patients with ischemic heart disease (IHD). Total subjects, *n* = 4317.

Age (Years)	Subjects with IHD Using TCMs		
	Males (%)	Females (%)	No. of Patients (%)
≤34	181 (9.03)	321 (13.89)	502 (11.63)
35–49	426 (21.25)	579 (25.04)	1005 (23.28)
50–64	673 (33.57)	722 (31.23)	1395 (32.31)
≥65	725 (36.16)	690 (29.84)	1415 (32.78)
Total	2005	2312	4317

**Table 2:** The top 10 single TCMs prescribed for IHD in Taiwan during 2000–2017. Total prescription number, *n* = 22,441.

Single TCM	Botanical Name	No. of Prescription	Percentage (%)
Danshen	*Salvia miltiorrhiza* Bunge	6483	28.89
Xiebai	*Allium macrostemon* Bunge/*Allium chinense* G.Don	5557	24.76
Yujin	*Curcuma wenyujin* Y.H. Chen & C. Ling/*Curcuma kwangsiensis* S.G. Lee & C.F. Liang/*Curcuma longa* L./*Curcuma phaeocaulis* Valeton	4780	21.3
Honghua	*Carthamus tinctorius* L.	3871	17.25
Huangqi	*Astragalus membranaceus* (Fisch.) Bunge/*Astragalus membranaceus* (Fisch.) Bunge var. *mongholicus* (Bunge) P.K. Hsiao	2240	9.98
Gualouren	*Trichosanthes kirilowii* Maxim./*Trichosanthes rosthornii* Harms	2140	9.54
Yanhusuo	*Corydalis yanhusuo* W.T. Wang	2017	8.99
Chuanxiong	*Ligusticum chuanxiong* Hort.	1592	7.09
Gegen	*Pueraria lobata* (Willd.) Ohwi/*Pueraria thomsonii* Benth.	1494	6.66
Danzhuye	*Lophatherum gracile* Brongn.	1387	6.18

**Table 3:** The traditional use and evidence-based indications for each single TCM related to IHD.

Single TCM	Traditional Use	IHD-Related Evidence-Based Indications
Danshen	invigorating the circulation of blood	increasing coronary blood flow [10], vasodilation [11], anticoagulant [11], antiatherosclerosis [11]
Xiebai	promoting qi to disperse stagnation	reducing the acute myocardial ischemia damage [12], inhibit platelet aggregation [13]
Yujin	activating qi for resolving stagnation, clearing heart fire and cooling blood, together with normalizing gallbladder to cure jaundice	activating blood circulation and removing blood stasis [14], improving myocardial metabolism [15]
Honghua	activating blood circulation, clearing blood stasis, and relieving pain	vasodilation [16], anticoagulant [17], antiatherosclerosis [18], reducing the myocardial ischemia injury [19]
Huangqi	invigorating qi and enriching the blood	vasodilation [20], promoting heart contraction [21], lowering blood pressure [22]
Gualouren	clearing heat and phlegm, dispelling lumps, moistening the intestines and bowels	none
Yanhusuo	promoting the circulation of blood, invigorating qi, relieving pain	protecting against myocardial ischemia injury [23], antiatherosclerosis [24]
Chuanxiong	invigorating the circulation of blood	vasodilation [25], anticoagulant [25], antiatherosclerosis [25], cardioprotective effect [25]
Gegen	relieving muscular spasm	increasing coronary blood flow [26], antiatherosclerosis [27], anticoagulant [28]
Danzhuye	clearing heat, diuresis	none

**Table 4:** The most prescribed 10 TCM formula for IHD in Taiwan during 2000–2017 (total prescription number, *n* = 22,441).

TCM Formula	Ingredients	No. of Prescription	Percentage (%)
Zhi-Gan-Cao-Tang	*Glycyrrhiza uralensis* Fisch./*Glycyrrhiza inflata* Batalin/*Glycyrrhiza glabra* L., *Panax ginseng* C.A. Mey., *Cinnamomum cassia* (L.) J. Presl, *Zingiber officinale* Roscoe, *Equus asinus* L., *Rehmannia glutinosa* Libosch., *Ophiopogon japonicus* (L.f.) Ker Gawl., *Cannabis sativa* L., *Ziziphus jujube* Mill.	5557	24.76
Xue-Fu-Zhu-Yu-Tang	*Angelica sinensis* (Oliv.) Diels, *Rehmannia glutinosa* Libosch., *Prunus persica* (L.) Batsch/*Prunus davidiana* (Carrière) Franch., *Carthamus tinctorius* L., *Citrus aurantium* L., *Paeonia lactiflora* Pall./*Paeonia veitchii* Lynch, *Bupleurum chinese* DC./*Bupleurum scorzonerifolium* Willd., *Glycyrrhiza uralensis* Fisch./*Glycyrrhiza inflata* Batalin/*Glycyrrhiza glabra* L., *Platycodon grandifloras* (Jacq.) A.DC., *Ligusticum chuanxiong* Hort., *Achyranthes bidentata* Blume.	4780	21.30
Sheng-Mai-Yin	*Panax ginseng* C.A. Mey., *Ophiopogon japonicus* (L.f.) Ker Gawl., *Schisandra chinensis* (Turcz.) Baill./*Schisandra sphenanthera* Rehder et E.H. Wilson.	3871	17.25
Gua-Lou-Xie-Bai-Ban-Xia-Tang (Gualou-Xiebai-Banxia decoction)	*Trichosanthes kirilowii* Maxim./*Trichosanthes rosthornii* Harms, *Allium macrostemon* Bunge/*Allium chinense* G. Don, *Pinellia ternata* (Thunb.) Makino, liquor	2240	9.98
Tian-Wang-Bu-Xin-Dan	*Panax ginseng* C.A. Mey., *Poria cocos* (Schwein.) F.A. Wolf, *Scrophularia ningpoensis* Hemsl., *Salvia miltiorrhiza* Bunge, *Polygala tenuifolia* Willd./*Polygala sibirica* L., *Platycodon grandifloras* (Jacq.) A.DC., *Angelica sinensis* (Oliv.) Diels, Schisandra chinensis (Turcz.) Baill./Schisandra sphenanthera Rehder et E.H. Wilson, *Ophiopogon japonicus* (L.f.) Ker Gawl., *Platycladus orientalis* (L.) Franco, *Ziziphus jujuba* Mill. Var. *spinosa* (Bunge) Hu ex H.F. Chow, *Rehmannia glutinosa* Libosch.	2140	9.54
Yang-Xin-Tang	*Astragalus membranaceus* (Fisch.) Bunge/*Astragalus membranaceus* (Fisch.) Bunge var. *mongholicus* (Bunge) P.K. Hsiao, *Poria cocos* (Schwein.) F.A. Wolf, *Pinellia ternata* (Thunb.) Makino, *Ligusticum chuanxiong* Hort., *Angelica sinensis* (Oliv.) Diels, *Polygala tenuifolia* Willd./*Polygala sibirica* L., *Ziziphus jujuba* Mill. var. *spinosa* (Bunge) Hu ex H.F. Chow, *Cinnamomum cassia* (L.) J. Presl, *Platycladus orientalis* (L.) Franco, *Schisandra chinensis* (Turcz.) Baill., *Panax ginseng* C.A. Mey., *Glycyrrhiza uralensis* Fisch./*Glycyrrhiza inflata* Batalin/*Glycyrrhiza glabra* L., *Zingiber officinale* Roscoe, *Ziziphus jujuba* Mill.	1494	6.66
Zhen-Wu-Tang	*Poria cocos* (Schwein.) F.A. Wolf, *Paeonia lactiflora* Pall., *Atractylodes macrocephala* Koidz., *Aconitum carmichaelii* Debeaux, *Zingiber officinale* Roscoe.	1297	5.78
Jia-Wei-Xiao-Yao-San	*Angelica sinensis* (Oliv.) Diels, *Poria cocos* (Schwein.) F.A.Wolf, *Gardenia jasminoides* J.Ellis, *Mentha canadensis* L. (M. haplocalyx Briq.), *Paeonia lactiflora* Pall., *Bupleurum chinense* DC./*Bupleurum scorzonerifolium* Willd., *Glycyrrhiza uralensis* Fisch./*Glycyrrhiza inflata* Batalin/*Glycyrrhiza glabra* L., *Atractylodes macrocephala* Koidz., *Paeonia suffruticosa* Andrews, *Zingiber officinale* Roscoe.	1199	5.34
Fufang-Danshen-Pian	*Salvia miltiorrhiza* Bunge, *Panax notoginseng* (Burkill) F.H.Chen, *Cinnamomum camphora* (L.) J. Presl	1024	4.56
Ji-Sheng-Shen-Qi-Wan	*Rehmannia glutinosa* Libosch., *Dioscorea opposita* Thunb./*Dioscorea doryophora* Hance/*Dioscorea japonica* Thunb., *Cornus officinalis* Siebold et Zucc., *Alisma plantago-aquatica* L. subsp. *orientale* (Sam.) Sam., *Poria cocos* (Schwein.) F.A. Wolf, *Paeonia suffruticosa* Andrews, *Cinnamomum cassia* (L.) J. Presl, *Aconitum carmichaelii* Debeaux, *Achyranthes bidentata* Blume, *Plantago asiatica* L./*Plantago depressa* Willd.	905	4.03

**Table 5:** The traditional use and evidence-based indications for each TCM formula related to IHD.

TCM Formula	Traditional Use	IHD-Related Evidence-Based Indications
Zhi-Gan-Cao-Tang	invigorating qi and enriching the blood, stabilizing heart rhythm	improving the myocardial contractility level, improving cardiac function [29], alleviating the oxidative stress of cardiomyocytes [30,31]
Xue-Fu-Zhu-Yu-Tang	invigorating the circulation of blood, promoting qi	antiatherosclerosis [32], anti-hypercholesterolemia [33], protection against ischemic stroke [34], protective effect against the myocardial apoptosis [34]
Sheng-Mai-Yin	supplementing qi and nourishing the vital essence, cardiac tonifying	reducing early ventricular contraction and paroxysmal tachycardia [29], improving the exercise tolerance of the cardiac recovery [35]
Gua-Lou-Xie-Bai-Ban-Xia-Tang (Gualou-Xiebai-Banxia decoction)	promoting qi to clear up yang, expelling phlegm and dispelling congestion	reducing ischemic myocardial impairment [36], protecting against the myocardial apoptosis in coronary heart disease [37], protecting against type II diabetes with acute myocardial ischemia [38]
Tian-Wang-Bu-Xin-Dan	tonifying blood, calming mind	none
Yang-Xin-Tang	nourishing heart and blood, calming mind	effecting plasma metabolism of patients with unstable angina [39]
Zhen-Wu-Tang	warming yang for diuresis	none
Jia-Wei-Xiao-Yao-San	dispersing stagnated liver qi for relieving qi stagnation, clearing heat and nourishing blood	none
Fufang-Danshen-Pian	promoting qi and activating blood	protecting cardiomyocytes against ischemia injury [40]
Ji-Sheng-Shen-Qi-Wan	consolidating renal essence, cardiac tonifying	none

**Table 6:** The most common two-drug combinations of TCM in a single prescription for IHD (Total prescription number, *n* = 22,441).

Chinese Herbal Formula or Drugs		No. of Prescriptions	Percentage (%)
First	Second		
Danshen	Xue-Fu-Zhu-Yu-Tang	1686	7.51
Zhi-Gan-Cao-Tang	Gua-Lou-Xie-Bai-Ban-Xia-Tang	1570	7.00
Danshen	Sheng-Mai-Yin	1459	6.50
Danshen	Zhi-Gan-Cao-Tang	1403	6.25
Danshen	Yujin	867	3.86
Zhi-Gan-Cao-Tang	Tian-Wang-Bu-Xin-Dan	840	3.74
Zhi-Gan-Cao-Tang	Xue-Fu-Zhu-Yu-Tang	818	3.65
Zhi-Gan-Cao-Tang	Yang-Xin-Tang	802	3.57
Xiebai	Gualouren	784	3.49
Danshen	Xiebai	782	3.48

**Table 7:** The most common three-drug combinations of TCM in a single prescription for IHD (total prescription number, *n* = 22,441).

Chinese Herbal Formula or Drugs			No. of Prescriptions	Percentage (%)
First	Second	Third		
Gua-Lou-Xie-Bai-Ban-Xia-Tang	Yang-Xin-Tang	Zhi-Gan-Cao-Tang	625	2.79
Tian-Wang-Bu-Xin-Dan	Gua-Lou-Xie-Bai-Ban-Xia-Tang	Zhi-Gan-Cao-Tang	589	2.62
Yang-Xin-Tang	Tian-Wang-Bu-Xin-Dan	Zhi-Gan-Cao-Tang	449	2.00
Tian-Wang-Bu-Xin-Dan	Yang-Xin-Tang	Gua-Lou-Xie-Bai-Ban-Xia-Tang	447	1.99
Danshen	Xue-Fu-Zhu-Yu-Tang	Sheng-Mai-Yin	345	1.54
Danshen	Gualouren	Xiebai	287	1.28
Danshen	Huangqi	Sheng-Mai-Yin	280	1.25
Danshen	Xue-Fu-Zhu-Yu-Tang	Zhi-Gan-Cao-Tang	270	1.20
Danshen	Sheng-Mai-Yin	Zhi-Gan-Cao-Tang	270	1.20
Danshen	Maidong [*Ophiopogon japonicus* (Thunb.) Ker Gawl.]	Wuweizi [*Schisandra chinensis* (Turcz.) Baill.]	257	1.15

## Data Availability

The data presented in this study are openly available in FigShare.
